# Early events during human coronavirus OC43 entry to the cell

**DOI:** 10.1038/s41598-018-25640-0

**Published:** 2018-05-08

**Authors:** Katarzyna Owczarek, Artur Szczepanski, Aleksandra Milewska, Zbigniew Baster, Zenon Rajfur, Michal Sarna, Krzysztof Pyrc

**Affiliations:** 1 Microbiology Department, Faculty of Biochemistry, Biophysics and Biotechnology, Jagiellonian University, Gronostajowa 7, 30-387 Krakow, Poland; 2 Virogenetics Laboratory of Virology, Malopolska Centre of Biotechnology, Jagiellonian University, Gronostajowa 7a, 30-387 Krakow, Poland; 3 Institute of Physics, Faculty of Physics, Astronomy and Applied Computer Sciences, Jagiellonian University, Lojasiewicza 11, 30-348 Krakow, Poland; 4 Department of Biophysics, Faculty of Biochemistry, Biophysics and Biotechnology, Jagiellonian University, Gronostajowa 7, 30-387 Krakow, Poland

## Abstract

The *Coronaviridae* family clusters a number of large RNA viruses, which share several structural and functional features. However, members of this family recognize different cellular receptors and exploit different entry routes, what affects their species specificity and virulence. The aim of this study was to determine how human coronavirus OC43 enters the susceptible cell. Using confocal microscopy and molecular biology tools we visualized early events during infection. We found that the virus employs caveolin-1 dependent endocytosis for the entry and the scission of virus-containing vesicles from the cell surface is dynamin-dependent. Furthermore, the vesicle internalization process requires actin cytoskeleton rearrangements. With our research we strove to broaden the understanding of the infection process, which in future may be beneficial for the development of a potential therapeutics.

## Introduction

There are currently six human coronaviruses described. The well-known human coronaviruses (HCoV) 229E and OC43 were described in 1960’s and for almost 40 years were considered to be the only representatives of *Coronaviridae* infecting humans. Emergence of Severe Acute Respiratory Syndrome–associated coronavirus (SARS-CoV) in 2002, followed by identification of HCoV-NL63 and HCoV-HKU1 revealed that these viruses are far more common and clinically relevant than previously expected. Further, emergence of the Middle East respiratory syndrome coronavirus (MERS-CoV) in 2012 proved that these pathogens frequently cross the species border and may pose a significant healthcare risk.

HCoV-OC43 infection has been associated with respiratory tract illnesses of varying severity^1^. The virus is considered to be the most common human coronavirus worldwide, with highest incidence during winter and spring months^1,2^. Due to genomic sequence similarities between HCoV-OC43, bovine coronavirus (BCoV) and, to a lesser extent, canine respiratory coronavirus, which cause the disease in respective animals, it has been assumed that zoonotic transmission to humans occurred relatively recently. The most recent common ancestor of HCoV-OC43 and BCoV has been dated to the end of 19^th^ century^3^ and the evolutionary rate was estimated to be 4 × 10^−4^ nucleotide changes per site per year^3^.

The coronavirus entry to the cell is a complex process, which requires a series of cellular factors. First, the virus binds to the attachment receptor. This interaction results in an increased cell surface density of virus particles and (or) facilitates interaction with the fusion receptor. To make an example, HCoV-OC43 and bovine coronavirus bind to N-acetyl-9-O-acetylneuraminic acid^4^, HCoV-HKU1 binds to O-acetylated sialic acids^5^, while HCoV-NL63 and SARS-CoV bind to heparan sulfate proteoglycans^6,7^. In some cases this step seems to be redundant^8^, while in others depletion of the adhesion receptor results in lack of interaction between the virus and the cell and consequently severe decrease in virus infectivity^6,9,10^. Nonetheless, the presence of the adhesion factor is not sufficient to make the cell permissive. Coronaviruses utilize a broad variety of fusion receptors. Most of the alphacoronaviruses use aminopeptidase N (CD13) for cell entry, with the exception of HCoV-NL63, which similarly to SARS-CoV employs human angiotensin-converting enzyme 2^11^. HCoV-OC43 was reported to utilize HLA class I molecule or sialic acids^12,13^, MERS-CoV - dipeptidyl peptidase 4 (DPP4 or CD26)^14^, whereas the receptor for HCoV-HKU1 remains unknown^5^. Recognition of different receptors implies not only different cellular tropism, but also different internalization routes. It is worth to mention, however, that recent reports also stress the importance of other cellular factors for virus tissue specificity, including tissue-specific proteases^15–18^.

The interaction with the receptor is only the beginning. The binding may induce fusion with cellular membranes, but in most of the cases this event is preceded by virus internalization *via* the endocytic route. The most common, and the best described route is clathrin-dependent endocytosis. This path is used by representatives of wide range of viral families (e.g., human enterovirus 71^19^, human metapneumovirus^20^, rabies virus^21^ and others). Upon receptor recognition, a viral particle is docked into a clathrin-coated pit. Its’ formation is initialized by concerted action of a protein complex that consists of FCHo1/2, Eps15 and intersectin-1. FCHo1/2 induces curvature of the plasma membrane and through Eps15 recruits Adaptor Protein 2 (AP2) to the nucleation site^22^. AP2 assembles clathrin units and once their concentration reaches a critical level, they polymerize to form a lattice on the membrane^23^. The structure deepens, stabilized by the cargo^24^. Budding of the vesicle is accompanied by a tubular neck formation, to which amphiphysin protein is attracted. It recruits dynamin, which polymerizes in a GTP dependent mode to finally cut off the cargo-containing vesicle from the cell surface^25,26^.

Another well-described path is caveolin-1 mediated endocytosis. Caveolae are flask-shaped cholesterol- and sphingolipid-rich smooth membrane invaginations stabilized with caveolin-1^27^. Loading of the caveolae with cargo results in recruitment of dynamin-2^28^, which cuts off the invagination, forming a neutral-pH vesicle called caveosome. The vesicle can be either transferred into Golgi complex, endoplasmic reticulum (ER) or progress to early endosomes^27^. Recently, besides these two canonical pathways, numerous alternative routes have been described, including entosis, flotillin-dependent entry, FEME, and IL2Rβ-like mechanisms.

The aim of this study was to map the entry of HCoV-OC43 to susceptible cell. At first, we confirmed that the virus binds to the cells and is internalized *via* endocytosis. Subsequently, we have shown that HCoV-OC43 particle after binding to the cell surface migrates to caveolae and is trafficked to endosomes by caveolin-mediated and dynamin-dependent route. Virus internalization requires unwinding of the actin cortex, yet actin filaments are not required for the entry.

## Results

### HCoV-OC43 enters HCT-8 the cell via endosomes

**Figure 1:**
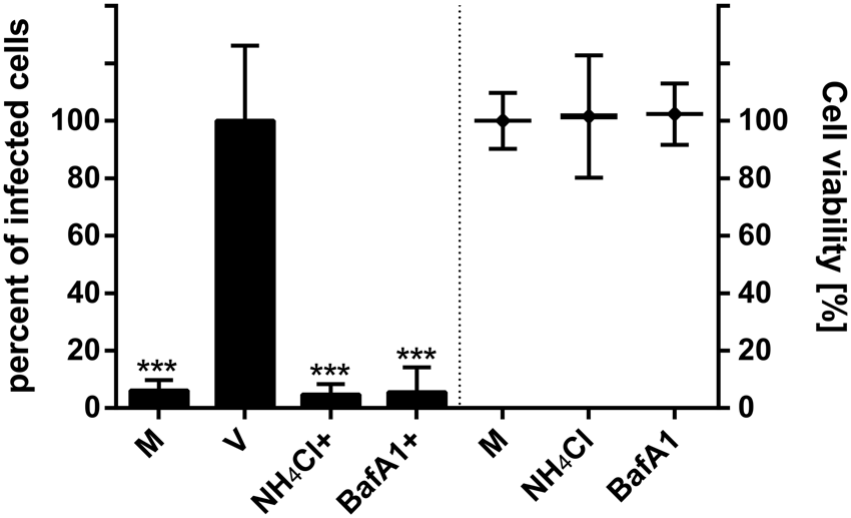
Inhibition of endosomal acidification with NH_4_Cl or bafilomycin A1 inhibits HCoV-OC43 infection in HCT-8 cell line. The infection efficiency determined with flow cytometry is expressed as the percentage of HCoV-OC43 infected cells, compared to the untreated control, and is presented on the left side of the graph. Right part of the graph shows the cell viability, as determined with an XTT assay. *NH*_4_*Cl* −50 mM NH_4_Cl; *BafA1* −2.5 nM bafilomycin A1; *control* – PBS; *M* – mock infected cells; *V* or *+* − HCoV-OC43 infected cells. The data is presented as the mean of a triplicate for each sample ± SD. To determine the significance of differences between compared groups, Single-Factor Analysis of Variance (ANOVA) was applied. ***P values < 0.05 were considered significant.

First, we determined whether the virus requires endocytosis, or the virus-cell fusion may occur on the cell surface. HCT-8 cells were pre-incubated with NH_4_Cl and subsequently incubated with the virus in the presence of NH_4_Cl, which prevents acidification of endosomes during maturation. Afterwards, media was refreshed to remove unbound virus particles. Infection was carried on for 3 days and its course was monitored with flow cytometry. As shown in Fig. 1, addition of NH_4_Cl early during the infection resulted in reduction of the number of virus-positive cells. Similarly, another inhibitor of endocytic compartment acidification, vacuolar-type ATPase inhibitor, bafilomycin A1, limited the number of infected cells (Fig. 1**)**.

**Figure 2:**
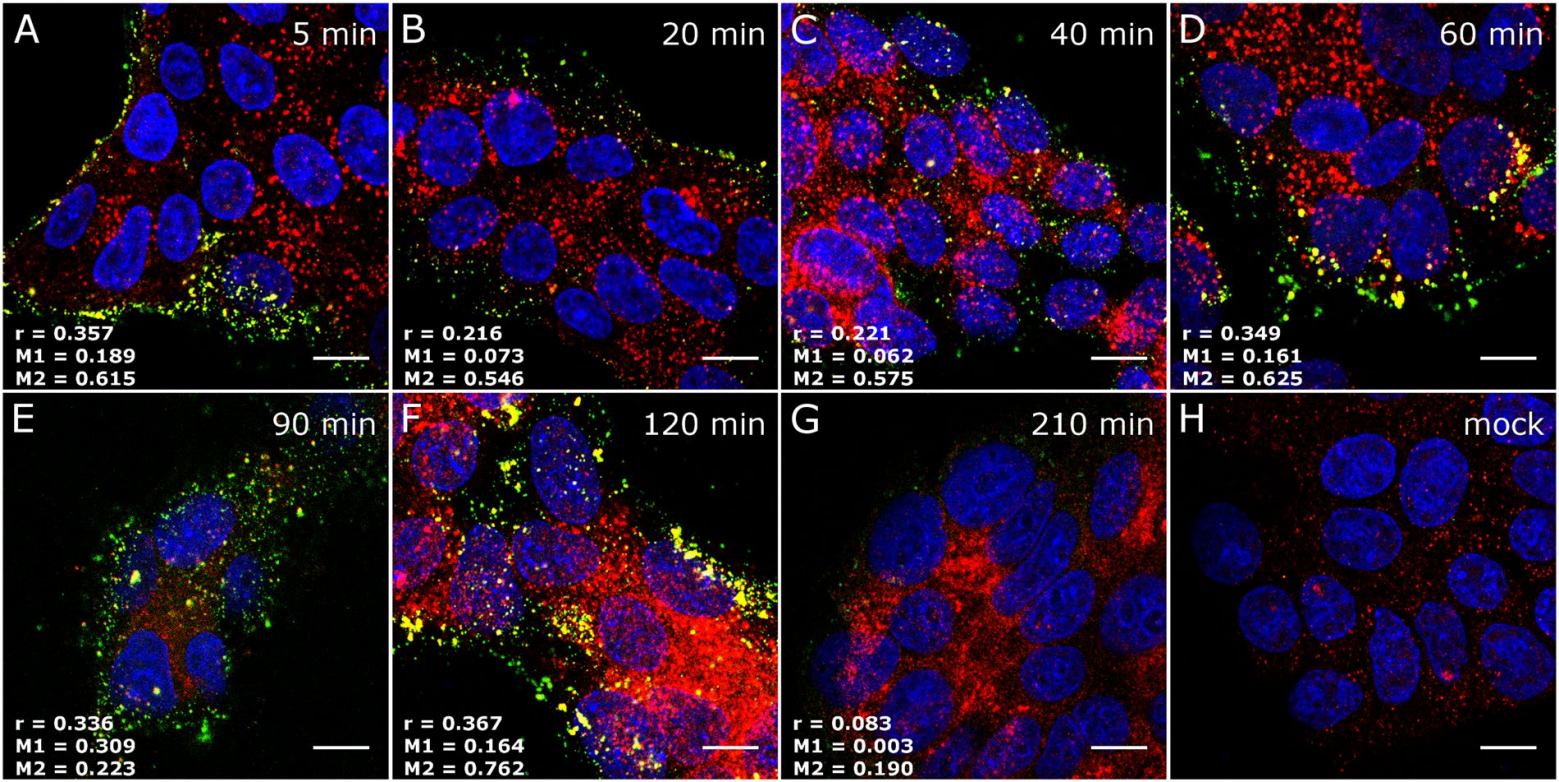
Co-localization of HCoV-OC43 with early endosomes’ marker EEA1. Co-localization of EEA1 with HCoV-OC43 in HCT-8 cells at different time points post infection was studied with confocal microscopy. Respective time points are indicated in the upper right corners. (**A**) HCoV-OC43-infected cells fixed 5 min p.i.; (**B**) 20 min p.i.; (**C**) 40 min p.i.; (**D**) 60 min p.i.; (**E**) 90 min p.i.; (**F**) 120 min p.i.; (**G**) 210 min p.i.; (**H**) mock-infected cells, stained with isotype control antibodies. The virus is visualized in green, EEA1 is shown in red, and nuclei are presented in blue. Scale bar = 10 μm. Co-localization parameters: r – Pearson’s coefficient; M1 - Manders’ coefficient M1 (EEA1 overlapping with the virus); M2 - Manders’ coefficient M2 (the virus overlapping with EEA1). The experiment was conducted at least thrice and representative images are presented.

In order to confirm our observation, we evaluated co-localization of virus particles with early endosome marker EEA1. HCT-8 cells were overlaid with HCoV-OC43 and incubated at 4 °C to synchronize entry of viral particles. Next, cultures were warmed up to 32 °C to enable intracellular transport and virus internalization. Subsequently, cultures were fixed, permeabilized and stained with specific antibodies to visualize viral proteins and EEA1 5-20 min post-inoculation (p.i). virus particles co-localized with EEA1, but remained in a close proximity to the cellular membrane (Fig. 2A,B). After that time virions started to gradually accumulate in larger, less abundant clusters (Fig. 2C–F) to eventually enter the cell.

### HCoV-OC43 particles migrate to the caveolin-1-rich invaginations

**Figure 3:**
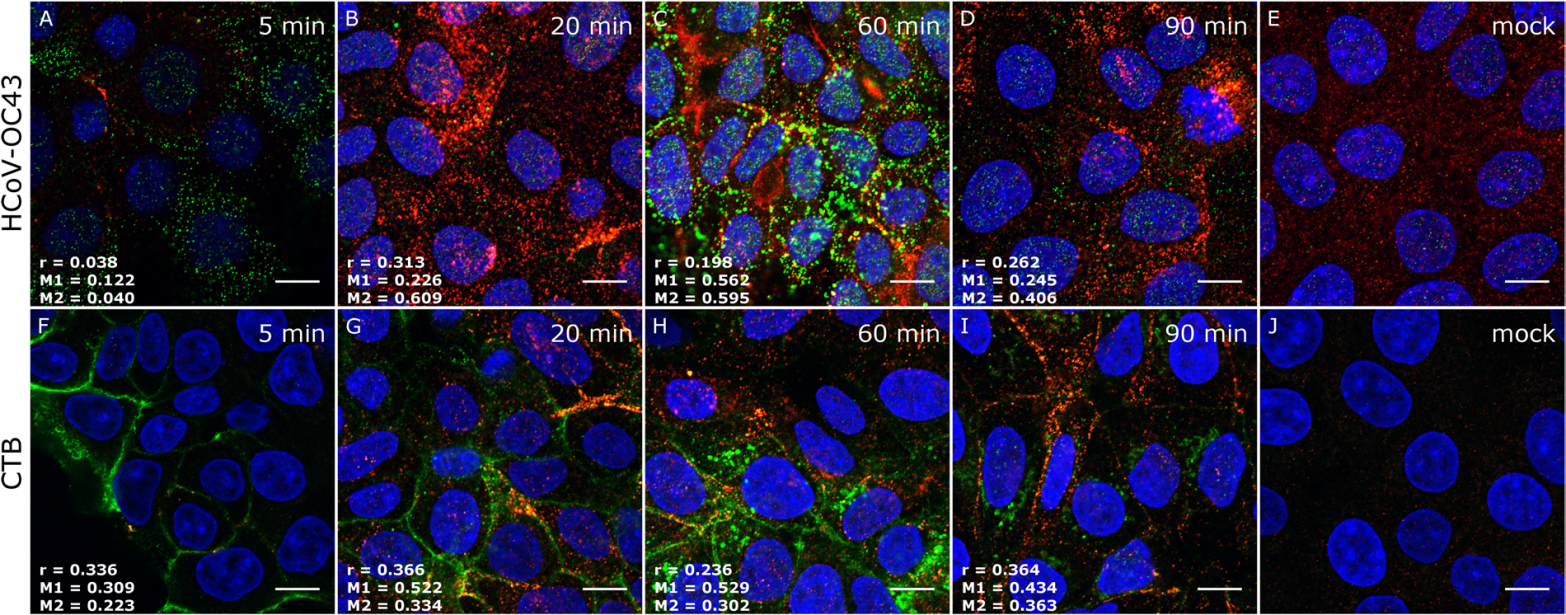
Co-localization of HCoV-OC43 with caveolin-1. Entry of HCoV-OC43 or cholera toxin B (CTB) was studied with confocal microscopy. Respective time points are indicated in the upper right corners. (**A**) HCoV-OC43-infected cells fixed 5 min p.i.; (**B**) 20 min p.i.; (**C**) 60 min p.i.; (**D**) 90 min p.i.; (**E**) mock-infected control cells; (**F**) CTB-overlaid cells fixed 5 min post-inoculation; (**G**) 20 min post-inoculation; (**H**) 60 min post-inoculation; (**I**) 90 min post-inoculation; (**J**) not-overlaid control cells. The virus and CTB are visualized in green, caveolin-1 is shown in red, and nuclei are presented in blue. Scale bar = 10 μm. Co-localization parameters: r – Pearson’s coefficient; M1 - Manders’ coefficient M1 (caveolin-1 overlapping with the virus); M2 - Manders’ coefficient M2 (the virus overlapping with caveolin-1). The experiment was conducted at least thrice, and representative images are presented.

To determine which endocytic route is employed, co-localization of virions with markers of different pathways was tested. Clearly, 5–90 min p.i. the virus co-localized with caveolin-1 (Fig. 3A–D). Prolonged co-localization is consistent with the reported kinetics of caveolae transport^29,30^. No co-localization with clathrin was noted (Supplementary Fig. 2). In order to validate this observation, cholera toxin B (CTB) conjugated with fluorescein isothiocyanate (FITC) was used as a positive control for caveolin-1 dependent entry^31,32^ (Fig. 3F–I).

### Disruption of caveolae hampers HCoV-OC43 entry

**Figure 4:**
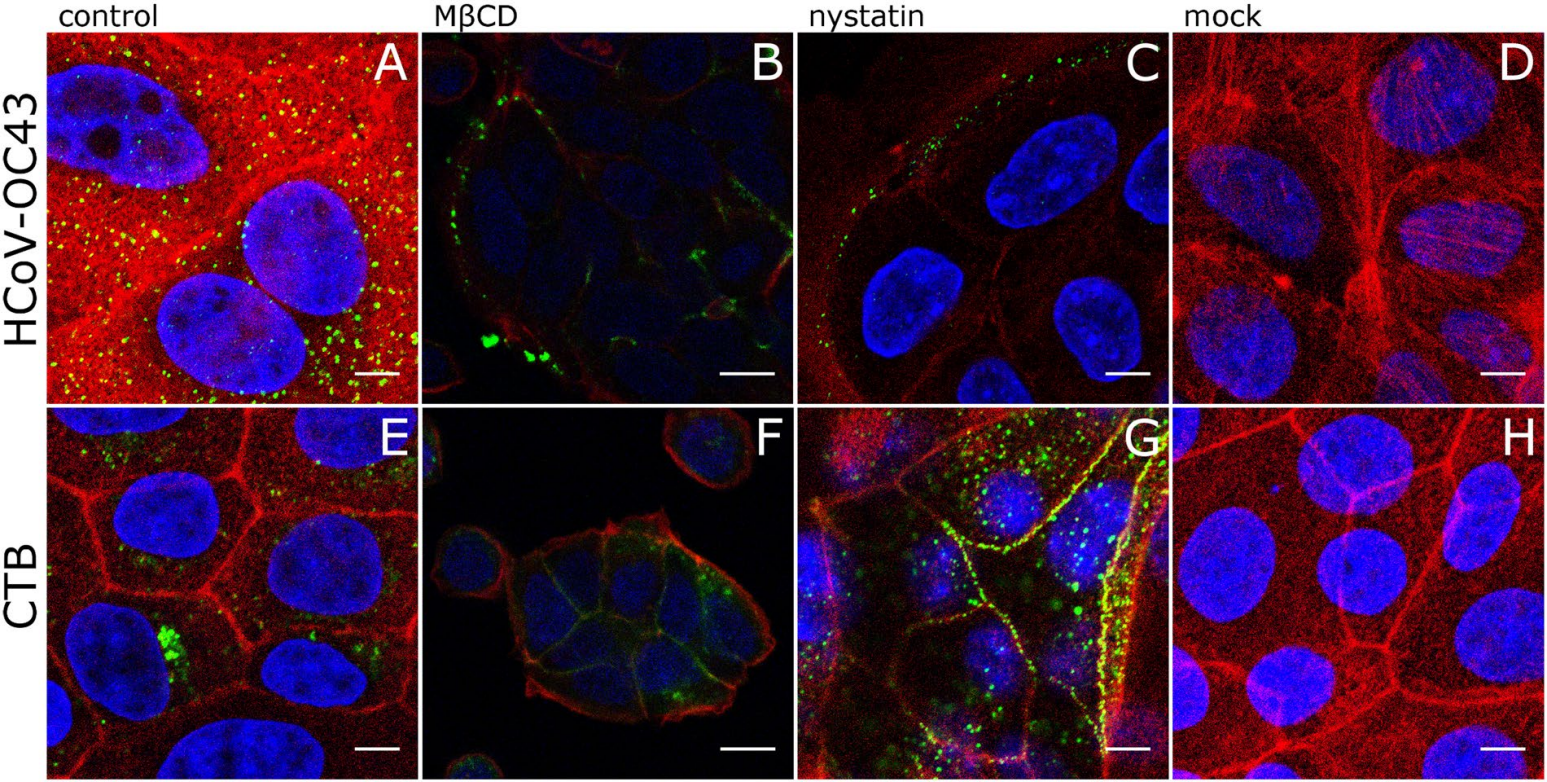
Disruption of caveolae with MβCD and nystatin hampers HCoV-OC43 entry. Confocal analysis of HCoV-OC43 or cholera toxin (CTB) entry to HCT-8 cells in the presence of cholesterol-sequestring agents was conducted. Following 1 h pretreatment with nystatin (100 μg/ml) or MβCD (100 μM), the cells were infected with HCoV-OC43 or overlaid with FITC-conjugated CTB (green). Actin cytoskeleton was stained to show the cell boundaries (red). Nuclei are shown in blue. (**A**) HCoV-OC43-infected inhibitor-untreated cells; (**B**) HCoV-OC43-infected MβCD-treated cells; (**C**) HCoV-OC43-infected nystatin-treated cells; (**D**) mock infected, inhibitor-untreated cells; (**E**) CTB inoculated, inhibitor-untreated cells; (**F**) CTB inoculated, MβCD-treated cells; (**G**) CTB inoculated, nystatin-treated cells; (**H**) mock inoculated, inhibitor-untreated cells. *mock* – mock inoculated cells; *control* – HCoV-OC43/CTB inoculated cells, in the absence of inhibitors. Scale bar = 10 μm. The experiment was conducted at least thrice and representative images are presented.

**Figure 5:**
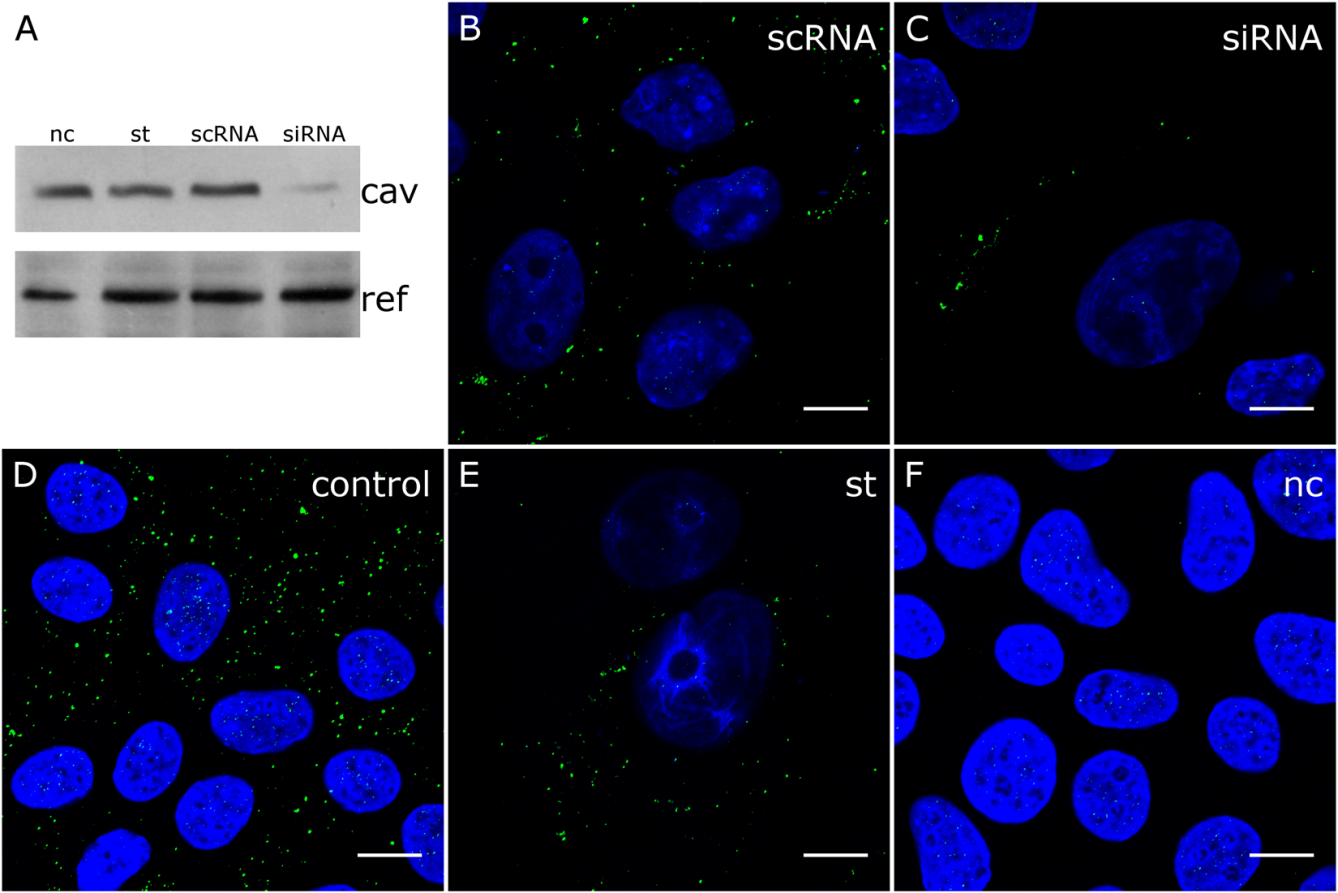
Inhibition of HCoV-OC43 entry to the caveolin-1 depleted cells. (**A**) Western blot analysis of the efficiency of siRNA-dependent caveolin-1 silencing (caveolin-1 expression in HCT-8 cells compared to β-tubulin expression in these cells). Confocal analysis of HCoV-OC43 localization 1 h p.i. in HCT-8 cells. *scRNA* (**B**) HCoV-OC43-infected, scrambled siRNA transfected cells; *siRNA* (**C**) HCoV-OC43-infected, caveolin-1-specific siRNA transfected cells; *control* (**D**) HCoV-OC43-infected, non-transfected cells; *st* (**E**) HCoV-OC43-infected, sham-transfected cells; *nc* (**F**) mock-infected, non-transfected cells. HCoV-OC43 is visualized in green and nuclei are shown in blue. Scale bar = 10 μm. The experiment was conducted at least twice, and representative images are presented.

In order to ensure that HCoV-OC43 enters the cell using caveolae, specific inhibitors of this endosomal pathway were used. Caveolae are formed in membrane clusters, where caveolin-1 is accompanied by cholesterol and sphingolipids. Consequently, the caveolin-mediated entry is sensitive to cholesterol-binding or depleting agents such as nystatin or methyl-β-cyclodextrin (MβCD). HCT-8 cells were pre-incubated with the compounds and overlaid with the virus. Following the incubation, cells were fixed, permeabilized and immunostained for HCoV-OC43 and actin, and virus entry was evaluated using confocal microscopy. As shown in Fig. 4, incubation with MβCD and nystatin (Fig. 4B,C) resulted in significant retention of virus on the cell surface, further proving that caveolae are required for virus internalization. Cholera toxin was used as a reference (Fig. 4F,G), as it was previously reported to enter the cell *via* caveosomes^31,32^. The MβCD-mediated inhibition of HCoV-OC43 entry (Fig. 4B) was more effective than CTB (Fig. 4F). To ensure that the observed effect is specific, we silenced expression of caveolin-1 in HCT-8 cells using siRNAs delivered in two consecutive transfections. Scrambled siRNAs were used as controls. Twenty-four hours after the second transfection, cells were incubated with HCoV-OC43 for 1 h. Subsequently, cells were fixed, permeabilized and immunostained to visualize the virus. In the cells transfected with caveolin-1 specific siRNAs protein levels were reduced by almost 80%, as assessed by Western blot, while scrambled siRNAs didn’t influence the protein level. β-tubulin was used as a reference household gene (Fig. 5A; the original image of the membrane is provided in Supplementary Fig. 1). Virus internalization to cells depleted of caveolin-1 was evaluated by confocal microscopy. As shown in Fig. 5, viral particles were retained on the cell surface in cells depleted of caveolin-1 (Fig. 5C), while no such effect was observed in control cells (Fig. 5B,D,E).

### Caveolae are required for HCoV-OC43 infection

**Figure 6:**
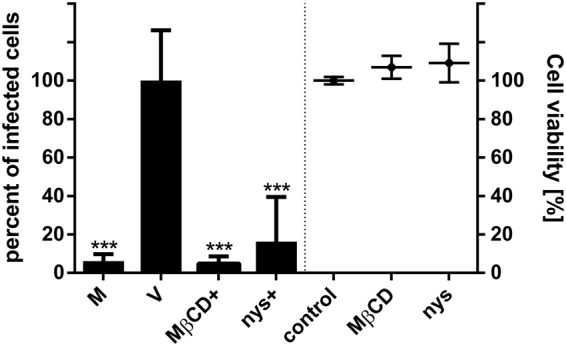
Inhibition of HCoV-OC43 infection in HCT-8 cells by MβCD and nystatin. HCT-8 cells pre-treated with cholesterol-sequestering agents were infected with HCoV-OC43 and analysed by flow cytometry 3 days p.i. The infection efficiency determined with flow cytometry is expressed as the percentage of HCoV-OC43 infected cells, compared to the untreated control, and is presented on the left side of the graph. Right part of the graph shows the cell viability, as determined with an XTT assay. *MβCD* −5 mM MβCD treated cells; *nys* −10 μg/ml nystatin treated cells; *control* − PBS treated cells; *M* – mock infected cells; *V* or *+* − HCoV-OC43 infected cells. The data is presented as the mean of a triplicate for each sample ± SD. To determine the significance of differences between compared groups, Single-Factor Analysis of Variance (ANOVA) was applied. ***P values < 0.05 were considered significant.

To test whether caveolin-mediated endocytosis is the major route of entry, cells were pre-incubated with MβCD or nystatin for 1 h and subsequently infected in the presence of inhibitors for 3 h. Subsequently, medium was discarded to remove unbound virus particles and fresh medium was applied. Infection was carried on for 3 days and its course was monitored with flow cytometry. Obtained results clearly show that MβCD and nystatin (Fig. 6) significantly affected virus infection in HCT-8 cells. In order to ensure that the observed effect does not result from cytotoxicity of inhibitors, cell viability was tested with XTT assay (Fig. 6).

### HCoV-OC43 entry is dynamin-dependent

**Figure 7:**
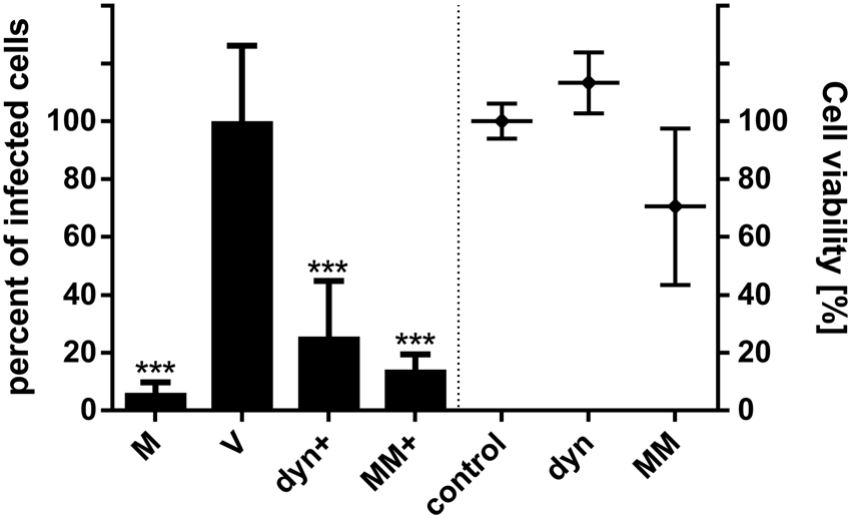
Inhibition of dynamin blocks HCoV-OC43 infection. HCT-8 cells were pre-treated with dynamin inhibitors, infected with HCoV-OC43 and analysed by means of flow cytometry 3 days p.i. The infection efficiency determined with flow cytometry is expressed as the percentage of HCoV-OC43 infected cells, compared to the untreated control, and is presented on the left side of the graph. Right part of the graph shows the cell viability, as determined with an XTT assay. *dyn* −40 μM dynasore; *MM* −10 μM MiTMAB; *control* – PBS treated cells; *M* −mock infected cells; *V* or *+ *− HCoV-OC43 infected cells. The data is presented as the mean of a triplicate for each sample ± SD. To determine the significance of differences between compared groups, Single-Factor Analysis of Variance (ANOVA) was applied. ***P values < 0.05 were considered significant.

To determine whether HCoV-OC43 entry process is dynamin-mediated, two different dynamin inhibitors were used: MiTMAB, which interacts with the lipid binding (PH) domain of dynamin and dynasore that non-competitively inhibits GTPase activity of dynamin^33^. Obtained results show that dynamin activity is required for effective HCoV-OC43 entry to the target cell (Fig. 7).

### HCoV-OC43 requires actin for successful entry

**Figure 8:**
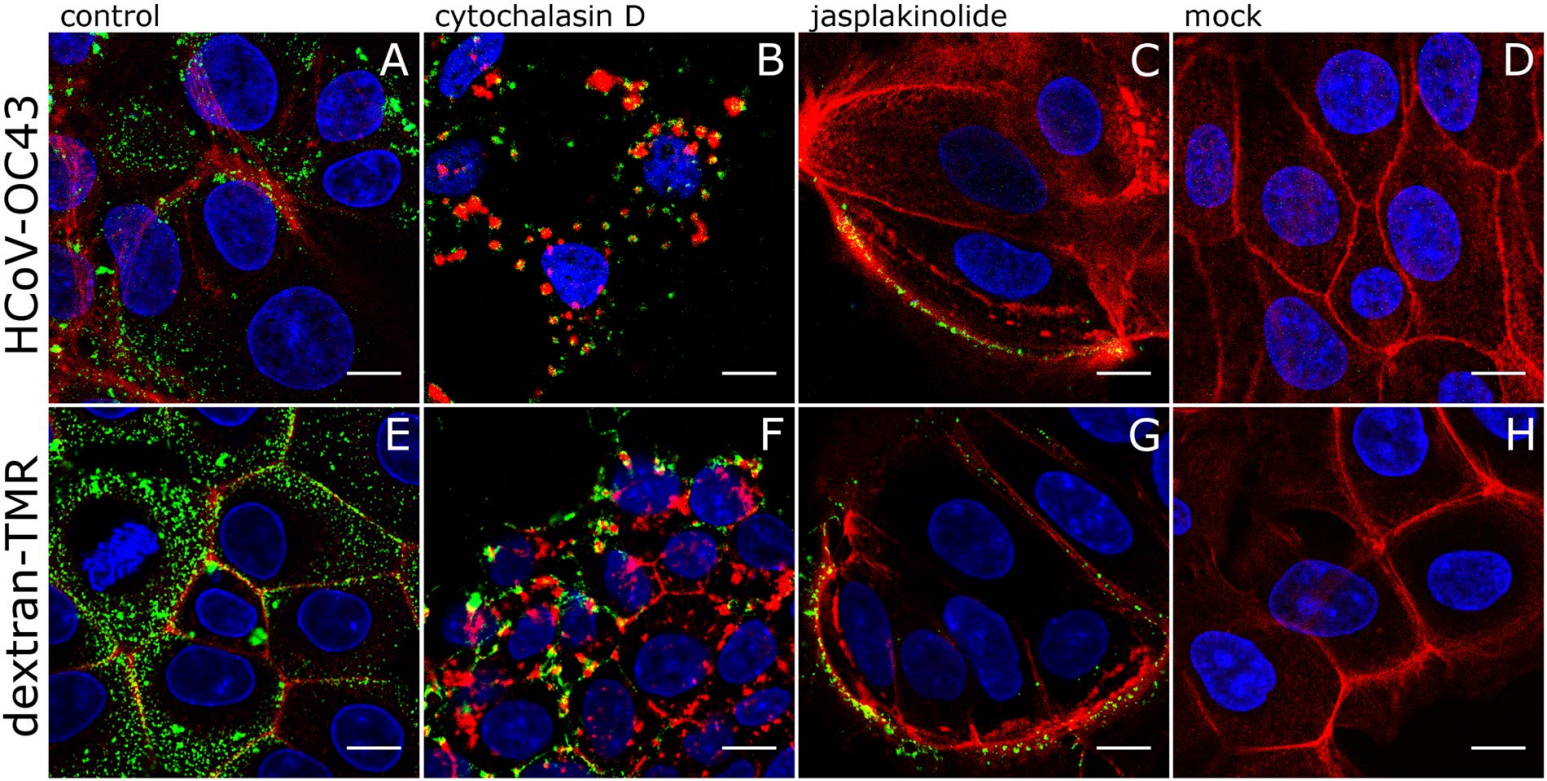
Inhibition of HCoV-OC43 infection in HCT-8 cells by compounds interfering with the actin cytoskeleton. HCoV-OC43 or dextran entry to the cell was studied using confocal microscopy. Following 1 h incubation of cells with inhibitors, cells were inoculated with HCoV-OC43 (**A**–**C**) or fluorescently labelled dextran (**E**–**G**) and incubated for 1 h. HCoV-OC43 and dextran are shown in green, actin cytoskeleton is presented in red. Nuclei are shown in blue. control HCoV-OC43/dextran-TMR inoculated inhibitor-untreated cells; *cytD* (**B,F**) HCoV-OC43/dextran-TMR inoculated, 2 μM cytochalazine D-treated cells; *jasp* (**C,G**) HCoV-OC43/dextran-TMR inoculated, 150 nM jasplakinolide-treated cells; *mock* (**D,H**) mock-inoculated inhibitor-untreated cells. Scale bar = 10 μm. The experiment was conducted at least thrice, and representative images are presented.

**Figure 9:**
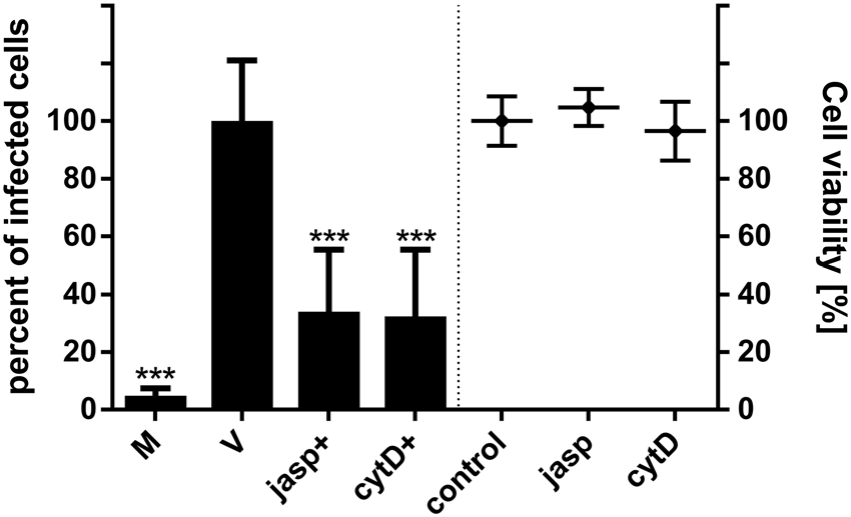
Inhibition of HCoV-OC43 infection of HCT-8 cells by agents interfering with the actin cytoskeleton. HCT-8 cells were pre-treated with actin inhibitors, infected with HCoV-OC43 and analysed by means of flow cytometry 3 days p.i. The infection efficiency determined with flow cytometry is expressed as the percentage of HCoV-OC43 infected cells, compared to the untreated control, and is presented on the left side of the graph. Right part of the graph shows the cell viability, as determined with an XTT assay. *jasp* −150 nM jasplakinolide; *cytD* −2 μM cytochalazine D; *control* – DMSO; *M* – mock infected cells; *V* or *+ *− HCoV-OC43 infected cells. The data is presented as the mean of a triplicate for each sample ± SD. To determine the significance of differences between compared groups, Single-Factor Analysis of Variance (ANOVA) was applied. ***P values < 0.05 were considered significant.

In order to study the role of cytoskeleton during virus entry, we used two compounds known to affect actin cytoskeleton. Briefly, HCT-8 cells were pre-treated with jasplakinolide or cytochalasin D, inoculated with HCoV-OC43 or dextran and further incubated in conditions allowing for intracellular transport (32 °C). Subsequently, cultures were fixed, permeabilized and stained with specific antibodies to visualize virus particles and actin cytoskeleton. Confocal imaging revealed that stabilization of actin cortex by jasplakinolide results in inhibition of HCoV-OC43 virus entry and dextran internalization (Fig. 8C,G). This may suggest that actin is important for vesicle formation, and the virus enters the cell by macropinocytosis. However, analysis of cells pre-treated with cytochalasin D revealed that depolymerization of actin filaments did not inhibit virus entry, but drastically modulated virus localization (Fig. 8B). Virus particle localized to non-structured actin deposits in the cell’s cytoplasm. On the other hand, dextran internalization was hampered (Fig. 8F). Both inhibitors drastically limited virus infection rate, as shown by flow cytometry (Fig. 9). No inhibition of virus entry or infection was observed for wortmannin, PI3K inhibitor known to hamper macropinocytosis (Supplementary Fig. 3).

### Re-directing HCoV-OC43 entry

**Figure 10:**
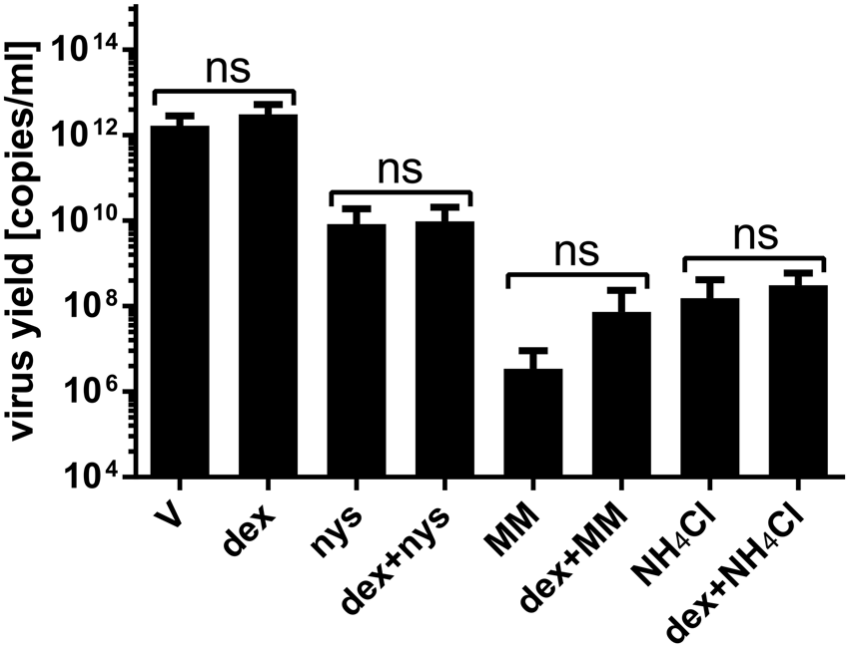
Macropinocytosis – stimulating agents re-direct virus trafficking in the cell. HCT-8 cells pre-treated with caveolin-1 and dynamin inhibitors were infected with HCoV-OC43 in presence or absence of dextran and viral yield was assessed by RT-qPCR 5 days p.i. *nys* −10 μg/ml nystatin; *MM* −10 μM MiTMAB; *NH*_4_*Cl* −50 mM NH_4_Cl; *V* – non-treated HCoV-OC43 infected cells; *dex* – dextran-TMR. The data is presented as the mean of a triplicate for each sample ± SD. To determine the significance of differences between compared groups, Single-Factor Analysis of Variance (ANOVA) was applied. ***P values < 0.05 were considered significant; ns – not significant.

Considering that micropinocytosis may be PI3K independent^34^, we made an effort to ensure that the virus does not use this pathway for entry. Briefly, we used dextran (70 kDa) as a cargo reported to enter the cell by micropinocytosis and we tested its co-localization with HCoV-OC43 virions during the virus entry^35^. As shown in Supplementary Fig. 4, strong co-localization of HCoV-OC43 with dextran was observed 5–210 min p.i. At the same time we observed vast decrease in HCoV-OC43 co-localization with EEA1 (early endosome’s marker), what suggested that in the presence of dextran the virus is internalized by a different pathway. This triggered another question, whether the dextran-induced micropinocytosis may initiate productive infection. To address this question, cells were infected with HCoV-OC43 in the presence of dextran and nystatin or MiTMAB or NH_4_Cl. Infection was carried on for 3 days and its course was monitored with flow cytometry. Obtained results (Fig. 10) clearly show that although the virus effectively enters the cell by macropinocytosis, it is not able to reach the replication site and to start productive infection. Inhibition of caveolin-dependent endocytosis in the presence of dextran did not block virus nor dextran internalization, but it blocked virus replication.

## Discussion

HCoV-OC43 remains incessantly one of the most important etiological factors for respiratory tract diseases in humans^1,2,36^. Considering lack of effective vaccine or therapeutics, and zoonotic potential of animal coronaviruses, understanding of the virus’ biology seems to be of importance.

The mode of entry remains unknown for a number of betacoronaviruses. One of the HCoV-OC43’s cousins, mouse hepatitis virus type 2 (MHV-2), undergoes clathrin-mediated endocytosis independent of Eps15^37^, while for SARS-CoV various pathways have been reported^38–40^. The internalization routes for the other three human betacoronaviruses, HCoV-OC43, HCoV-HKU1 and MERS-CoV, have not been described thus far. In this work we delineated early steps of HCoV-OC43 infection in human cells.

First, we aimed to map the events subsequent to virus-receptor interaction. For that we checked whether the virus is able to fuse with cellular membrane on the cell surface or it requires prior internalization. It is generally believed that during internalization additional stimuli is provided due to acidification of the microenvironment and in some cases pH-dependent activation of proteases. Consequently, viruses sensitive to inhibitors preventing pH decrease are believed to require endocytosis for entry. We showed that bafilomycin A1 and ammonium chloride strongly inhibit virus replication (Fig. 1). One may, however, question whether during prolonged incubation only virus entry is affected. To ensure validity of our observation, subcellular localization of viruses entering the cell was tested and its co-localization with endosomal markers was verified. Obtained results confirmed that virions entering the cell co-localize with EEA1 molecule (Fig. 2), which is an established marker of early endosomes.

Knowing that HCoV-OC43 enters the cell by endocytic route, we made an effort to delineate the mechanism of this process. For that, we tested whether virus co-localizes with common endocytic markers. Performed research revealed that interaction between the virus and the cell triggers recruitment of caveolin-1 and subsequent caveolae assembly (Fig. 3). Assembly of these structures depends on membrane content and flexibility, and we investigated the influence of compounds modifying cholesterol content/availability on HCoV-OC43 entry. Consistently, nystatin or MβCD pretreatment caused retention of the virus on the cell surface (Fig. 4). Both inhibitors blocked HCoV-OC43 infection, proving the relevance of this pathway for virus replication (Fig. 6). It is, however, known that chemical inhibitors may show non-specific effects^41–43^. In order to ensure that observed effect is not an artifact, caveolin-1 was depleted in HCT-8 cells using RNAi technology (Fig. 5). All experiments consistently showed that HCoV-OC43 entry is caveolin-1 dependent.

The vesicle and its cargo were tracked during trafficking to the replication site. First, we have shown that newly formed caveolae carrying HCoV-OC43 virions are cut off the cell surface membrane by dynamin (Fig. 7). Dynamin, a ring-like shaped GTPase driving vesicle scission, has been extensively studied in the context of clathrin-dependent endocytosis^44–46^, but it was also demonstrated to participate in caveolae trimming^47,48^.

Next, the role of cytoskeleton in virus transport was studied. Interestingly, modification of the actin dynamics affected virus entry, but actin was not essential for virus internalization. Disruption of the actin filaments did not result in inhibition of virus entry, but virions co-localized in the cytoplasm with unstructured actin deposits, suggesting interaction between virus-carrying vesicles and actin filaments (Fig. 8B). On the other hand, stabilization of actin cytoskeleton resulted in retention of virions on cell surface, but we believe that it may be linked with the physical barrier formed by stabilized actin cortex (Fig. 8C). Obtained results are consistent with literature data^49,50^.

Further, we have tested the co-localization of HCoV-OC43 with dextrans, which were previously reported to enter the cell by macropinocytosis^35^. To our surprise we recorded co-localization of these two cargoes (Supplementary Fig. 4). Subsequent examination revealed that in the presence of dextrans, which are also known inducers of macropinocytosis, the virus is switching the internalization route to macropinocytosis (Supplementary Fig. 4). It seems, however, that virus internalization by this route does not allow for effective entry and replication (Fig. 10). One may hypothesize that during micropinocytosis the pH in the vesicle is not decreased and the virus – cell fusion does not occur. In such a scenario, viruses are recycled to the cell surface or degraded intracellularly. Interestingly, it was shown for MHV and SARS-CoV that endocytosis may play a role in cell-to-cell spread and micropinocytosis^51^, but one may assume that it may differ between these species.

Our study on the early steps of HCoV-OC43 replication cycle was performed in HCT-8 cell line, which constitutes a conventional and accepted model for research on this coronavirus^52,53^. One may, however, question whether more natural system as human airway epithelium (HAE) culture wouldn’t be more appropriate. Unfortunately, the HAE culture does not support replication of the strain replicating *in vitro* and therefore it was not possible to compare these two models. This is a direct consequence of cell culture adaptation, as recently reported for laboratory strains of HCoV-OC43, HCoV-229E and HCoV-HKU1^54^. It was suggested that the presence of TMPRSS2 protease on the cell surface modifies the entry route allowing it to bypass the classical endocytic entry^55^. Similar conclusions were also drawn by others^56–58^, but we recently provided an alternative explanation for HCoV-NL63 virus^59^.

In conclusion, our results allow for understanding of the first steps of HCoV-OC43 infection. We have shown that following interaction with a receptor protein on the cell surface the virus enters the cell *via* caveolae and is transported along actin cytoskeleton. Interestingly, we have shown that even though there are alternative entry pathways for the virus, such event does not lead to the productive infection.

## Materials and Methods

### Cells and virus

HCT-8 cells (ATCC: CCL-244 Human ileocecal colorectal adenocarcinoma) were cultured in Dulbecco-modified Eagle’s medium (DMEM, Thermo Scientific) supplemented with 3% fetal bovine serum (FBS, Thermo Scientific), 100 U/ml penicillin, and 100 μg/ml streptomycin and 5 μg/ml ciprofloxacin. Cells were maintained in a 5% CO_2_ incubator at 37 °C.

HCoV-OC43 (ATCC: VR-1558) was propagated in HCT-8 cells in DMEM supplemented with 2% FBS, 100 U/ml penicillin, and 100 μg/ml streptomycin. Cells were lysed 5 days after infection by 2 freeze & thaw cycles and virus was titrated according to the Reed & Muench formula^60^. As a control, mock-infected HCT-8 cells were used. Virus and mock aliquots were stored at −80 °C.

### Inhibitors

Methyl-β-cyclodextrin (MβCD), nystatin (nys), bafilomycin A1 (bafA), cytochalasin D (cytD) and wortmannin (wort) were purchased from Sigma Aldrich. Dynasore (dyn) and MiTMAB (MM) were purchased from Abcam, jasplakinolide (jasp) from Merck, NH_4_Cl from Bioshop. All stock solutions were prepared either in DMSO (jasp) or PBS (MβCD, nys, BafA1, cytD, wort, dyn, MM and NH_4_Cl) and stored at 4 °C or −20 °C, according to the manufacturer’s recommendations.

### Virus titration

The titration assay was performed as described previously by Reed and Muench^60^. Briefly, confluent HCT-8 cells were cultured in 96-well plates. Serial five-fold dilutions of virus stock were prepared in DMEM supplemented with 2% FBS, 100 U/ml penicillin, and 100 μg/ml streptomycin, and 100 μl of the diluted virus was added into each well. The cells were incubated at 32 °C under 5% CO_2_ for 5 days and the cytopathic effect occurrence was scored using an inverted microscope. The number of wells with obvious cytopathic effect was counted and the TCID_50_ values were calculated according to the Reed–Muench formula.

### Co-localization assay

HCT-8 cells were seeded in the complete medium onto glass slides in 6-well plates. After 2 days cell culture medium was replaced with serum-free DMEM supplemented with 100 U/ml penicillin and 100 μg/ml streptomycin two hours prior to the experiment. Next, cells in each well were treated with 100 μl of HCoV-OC43 stock (or mock) and incubated for 1 h at 4 °C to synchronize viral particles entry from the cell surface. CTB conjugated to FITC (Sigma), diluted to the final concentration 40 μg/ml in DMEM supplemented with 2% FBS, 100 U/ml penicillin, and 100 μg/ml streptomycin, was used as a positive control for caveolin-dependent entry pathway.

Subsequently, cultures were transferred to 32 °C. At indicated in each experiment p.i. times, the cells were washed twice with PBS, fixed in cold 4% paraformaldehyde for at least 20 min at room temperature and immunostained for HCoV-OC43, caveolin-1 or EEA1.

### Immunofluorescence assay

The fixed cells were washed twice with PBS and permeabilized with 0.5% Triton X-100 for 13 min at room temperature. Afterwards, samples were blocked overnight at 4 °C in 5% bovine serum albumin (BSA) in PBS and incubated for 2 h at room temperature with primary anti-HCoV-OC43 antibodies (MAB9012, Merck) diluted 1:1000 in 3% BSA in PBS. Subsequently, samples were incubated for 1 h with Alexa Fluor 488 labeled goat anti-mouse IgG (Thermo Fisher Scientific) diluted 1:400 in 3% BSA in PBS. To visualize host cell proteins, the cells were blocked again overnight at 4 °C with 10% FBS in PBS, incubated for 2 h at room temperature with primary antibodies (Caveolin-1 N-20 Antibody, sc-894, Santa Cruz Biotechnology; Clathrin HC Antibody (C-20), sc-6579, Santa Cruz Biotechnology; EEA1 H-300 Antibody, sc-33585, Santa Cruz Biotechnology) diluted 1:100 in 2.5% FBS in PBS, and finally with Atto 633 labeled goat anti-rabbit IgG (Thermo Fisher Scientific) or Alexa Fluor 546 goat anti-rabbit (Thermo Fisher Scientific) diluted 1:200 in 2.5% FBS in PBS.

In experiments showing localization of virions in the cell actin cytoskeleton was visualized. Briefly, after HCoV-OC43 labelling, cells were stained with Atto 633-phalloidin (Thermo Fisher Scientific) diluted 1:50 in PBS for 1 h at room temperature. Nuclei were stained with DAPI (Thermo Fisher Scientific) diluted 1:10 000 in PBS.

After immunostaining in all the cases cells were washed with 0.5% TWEEN-20 in PBS. Finally, stained cultures were mounted on glass slides in ProLong Diamond antifade medium (Thermo Fisher Scientific) and stored at 4 °C.

### Visualization of HCoV-OC43 entry inhibition

Cells were seeded on glass slides in 6-well plates and cultured at 37 °C for 48 h. After that time media were removed and cells were incubated in DMEM with 2% FBS, 100 U/ml penicillin and 100 μg/ml streptomycin supplemented with endocytosis inhibitors at 37 °C for 1 h. Subsequently, media were removed and HCoV-OC43 stock was overlaid on the cells in the presence or absence of inhibitors and cultures were incubated at 32 °C for 1 h. Unbound virus particles were removed by rinsing the cells twice in PBS. Cells were with cold 4% paraformaldehyde for at least 20 min at room temperature and immunostained for HCoV-OC43 and actin.

### siRNA silencing

Pooled siRNAs targeting caveolin-1 (sc-44202) and scrambled siRNAs (sc-44237) were purchased from Santa Cruz Biotechnology. HCT-8 cells, cultured on glass slides in a 6-well plate for 1 day (80% confluent), were transfected with siRNA using Lipofectamine RNAiMAX (Thermo Fisher Scientific) according to the manufacturer’s instructions. The final amount of siRNA added to each well was 25 pmol. The procedure was repeated 24 h later to improve the silencing effect and further reduce caveolin-1 protein level in the transfected cells. HCoV-OC43 infection (with the viral stock of TCID_50_ = 2 300 000/ml) was carried out 24 h later at 32 °C for 1 h, cells were fixed and immunostained for HCoV-OC43, caveolin-1 and actin as described above. Concomitantly, on the infection day, caveolin-1 protein levels in test samples, mock-transfected and non-transfected cells were compared with Western blotting. β-tubulin was used as the control. Proteins were isolated using RIPA Buffer supplemented with 0.5 M EDTA and 1× proteinase inhibitor.

### Western blot analysis

Cell lysates were mixed 1:1 with 2 × SDS-PAGE sample buffer and incubated for 5 min at 95 °C. Afterwards, they were separated by SDS-PAGE electrophoresis and subsequently electrotransferred onto nitrocelulose membrane (Amersham). Membranes were blocked with 5% BSA (Bioshop) in Tris-Buffered Saline with Tween 20 (TBST). Membranes were incubated with primary antibodies (Caveolin-1 N-20 Antibody (sc-894, Santa Cruz Biotechnology) diluted 1:1000 in 2.5% BSA in TBST and β-tubulin Antibody (sc-134234, Santa Cruz Biotechnology) diluted 1:2000 in 2.5% BSA in TBST for 2 h and washed in TBST. Subsequently, membrane was incubated with HRP-labeled anti-rabbit IgG antibody (A0545, Sigma) diluted 1:20 000 in 1% BSA in TBST) for 1 h and finally the signal was developed using the ECL system (Amersham).

### Inhibition of HCoV-OC43 replication by endocytosis inhibitors

Cells were seeded in 96-well plates and cultured at 37 °C for 48 h. After that time media were removed and cells were incubated in DMEM with 2% FBS, 100 U/ml penicillin and 100 μg/ml streptomycin supplemented with endocytosis inhibitors at 37 °C for 1 h. Subsequently, media were removed and HCoV-OC43 stock (TCID_50_ = 800/ml) was overlaid on the cells in the presence or absence of inhibitors and cultures were incubated at 32 °C for 3 h. Unbound virus particles were removed by rinsing the cells thrice in PBS. The infected cells were further cultured in DMEM supplemented with 2% FBS, 100 U/ml penicillin, and 100 μg/ml streptomycin in the presence of inhibitors at 32 °C for 5 days. Finally, inhibitors’ cytotoxicity was inspected with XTT assay in mock-infected cells and viral RNA was isolated from cell culture medium. HCoV-OC43 yield was quantified by RT-qPCR.

### Flow cytometry (FACS) analysis

Three days post infection cells were harvested by trypsinization and pelleted in sterile PBS. After fixation in 4% paraformaldehyde, cells were blocked and immunostained for HCoV-OC43 as described above. Washed, resuspended in PBS cells were analyzed by flow cytometry (FACSCalibur, Becton Dickinson). Cell Quest software (Becton Dickinson) was used for data analysis. The infection rate was calculated relatively to untreated, HCoV-OC43 infected cells.

### Quantitative real time PCR

Virus detection and quantification was performed by reverse transcription reaction followed by quantitative real-time PCR (RT-qPCR). Viral nucleic acids were isolated from cell culture supernatants using Viral DNA/RNA Kit (A&A Biotechnology), according to the manufacturer’s protocol. Reverse transcription was carried out with High Capacity cDNA Reverse Transcription Kit (Thermo Fisher Scientific), according to the manufacturer’s protocol. Serially diluted pTZ57R/T plasmid carrying DNA HCoV-OC43 N gene served as standards. Concentration of the linearized form of the standard was assessed using a spectrophotometer and gel electrophoresis.

Subsequently, PCR was performed using KAPA PROBE FAST qPCR Master Mix (Kapa Biosystem), specific probe (TGA CAT TGT CGA TCG GGA CCC AAG TA) labeled with FAM (6-carboxyfluorescein) and TAMRA (6-carboxytetramethylrhodamine), and primers specific for HCoV-OC43 (forward: 5-AGC AAC CAG GCT GAT GTC AAT ACC-3, reverse: 5-AGC AGA CCT TCC TGA GCC TTC AAT-3). Rox was used as a reference dye. The amplification program was set at 50 °C for 2 min, 92 °C for 10 min, 40 cycles of 92 °C for 15 s, and 60 °C for 1 min.

### Cell viability assay (XTT assay)

HCT-8 cells were cultured on 96-well plates, as described above. Cell viability was examined using the XTT Cell Viability Assay (Biological Industries), according to the manufacturer’s protocol. Briefly, the medium was discarded and 70 μl of DMEM supplemented with 2% FBS, 100 U/ml penicillin, and 100 μg/ml streptomycin and 30 μl of the activated XTT solution was added to each well. After 2 h incubation at 37 °C, the medium was transferred onto a new 96-well plate and signal quantified at λ = 490 nm using the colorimeter (FlexStation Multi-Mode Microplate Reader, Molecular Devices). Experiments were performed at least 3 times. The obtained results were normalized to the control samples, where cell viability was set to 100%.

### Fluorescence and confocal microscopy

The fluorescent images were taken under a ZEISS LSM 710 (release version 8.1) confocal microscope with 40× oil immersion objective and acquired with ZEN 2012 SP1 (black edition, version 8.1.0.484) software. Stacks acquisition parameters were as follows: frame size 1024 × 1024, step size 0.15 μm, pixel size 0.06696 × 0.06696 µm. For image processing ImageJ FIJI (National Institutes of Health, Bethesda, Maryland, USA) version was used.

### Statistical analyses

All the experiments were performed at least 3 times. The data is presented as the mean of a triplicate for each sample ± SD. To determine the significance of differences between compared groups, Single-Factor Analysis of Variance (ANOVA) was applied. P values < 0.05 were considered significant.

### Data availability

All data generated or analysed during this study are included in this published article.

## Electronic supplementary material

Supplementary Figures
