# Supplementary material for: Early events during human coronavirus OC43 entry to the cell
Source: Sci Rep. 2018 May 8;8:7124. doi: 10.1038/s41598-018-25640-0 (PMC5940804; doi:10.1038/s41598-018-25640-0)
Supplement: Supplementary file 1 — Supplementary Figures [file 41598_2018_25640_MOESM1_ESM.pdf]

Early events during human coronavirus OC43 entry to the cell.

Katarzyna Owczarek[a,b], Artur Szczepanski[a,b], Aleksandra Milewska[a,b], Zbigniew Baster[c], Zenon Rajfur[c], Michal Sarna[b,d], Krzysztof Pyrc[a,b,*]

[a] Microbiology Department, Faculty of Biochemistry, Biophysics and Biotechnology, Jagiellonian University, Gronostajowa 7, 30-387 Krakow, Poland.
[b] Virogenetics Laboratory of Virology, Malopolska Centre of Biotechnology, Jagiellonian University, Gronostajowa 7a, 30–387 Krakow, Poland
[c] Institute of Physics, Faculty of Physics, Astronomy and Applied Computer Sciences, Jagiellonian University, Lojasiewicza 11, 30-348 Krakow, Poland.
[d] Department of Biophysics, Faculty of Biochemistry, Biophysics and Biotechnology, Jagiellonian University, Poland.

[*] **Corresponding author**

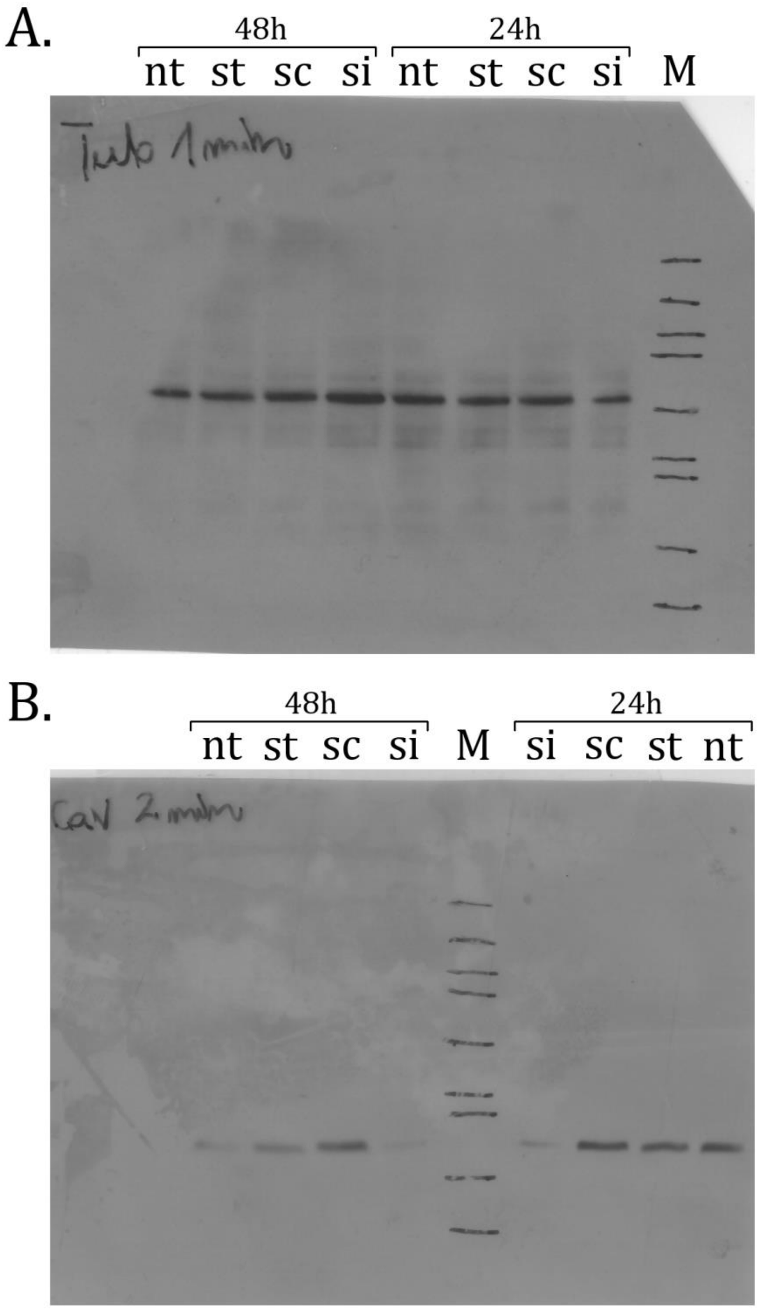

Supplementary Figure 1. Full-size Western blots from Figure 5. β-tubulin (**B**) or caveolin-1 (**A**) expression in HCT-8 cells after siRNA transfections. *nt* – non-transfected cells; st – sham-transfected cells; *sc* – scrambled siRNA transfected cells; *si* – caveolin-1- specific siRNA transfected cells; *M* – protein size marker, PageRuler Plus Prestained Protein Ladder (*Thermo Fisher Scientific*). 24 h – lysates of the transfected cells lysed 24 h after the second transfection; 48 h – lysates of the transfected cells lysed 48 h after the second transfection.

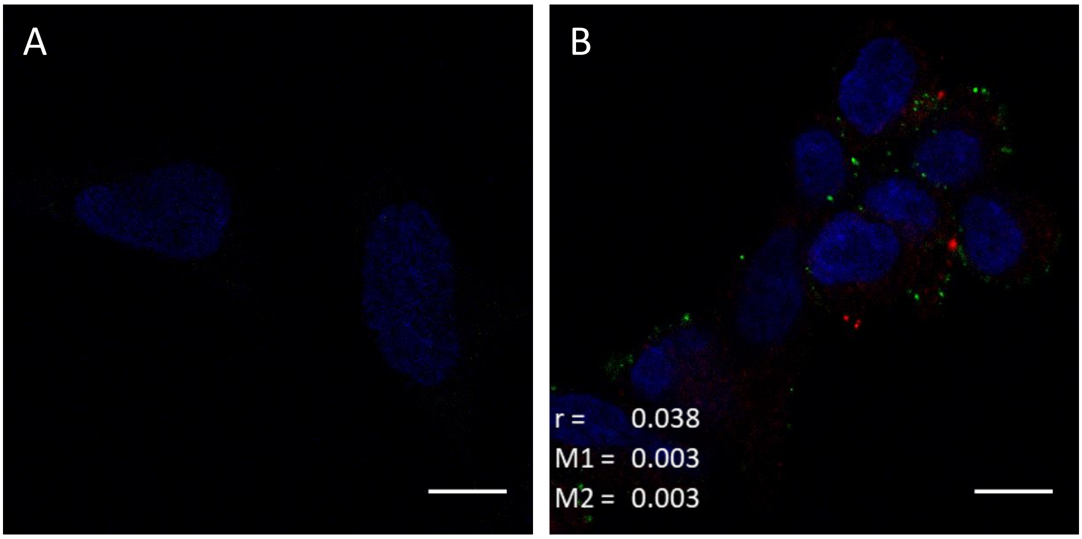

Supplementary Figure 2. Colocalization of HCoV-OC43 and clathrin in HCT-8 cells. Cells overlaid with virus were fixed 10 min post infection and co-localization between viral nucleocapsid protein and clathrin was studied by confocal microscopy. The virus is shown in green, clathrin in red, and nuclei in blue. A – mock-infected cells with primary antibody isotype control staining. B – HCoV-OC43-infected cells stained for clathrin. Scale bar = 10 µm. Co-localization parameters: r – Pearson’s coefficient; M1 - Manders' coefficient M1 (EEA1 overlapping with the virus); M2 - Manders' coefficient M2 (the virus overlapping with clathrin). The experiment was conducted at least thrice, and representative images are presented.

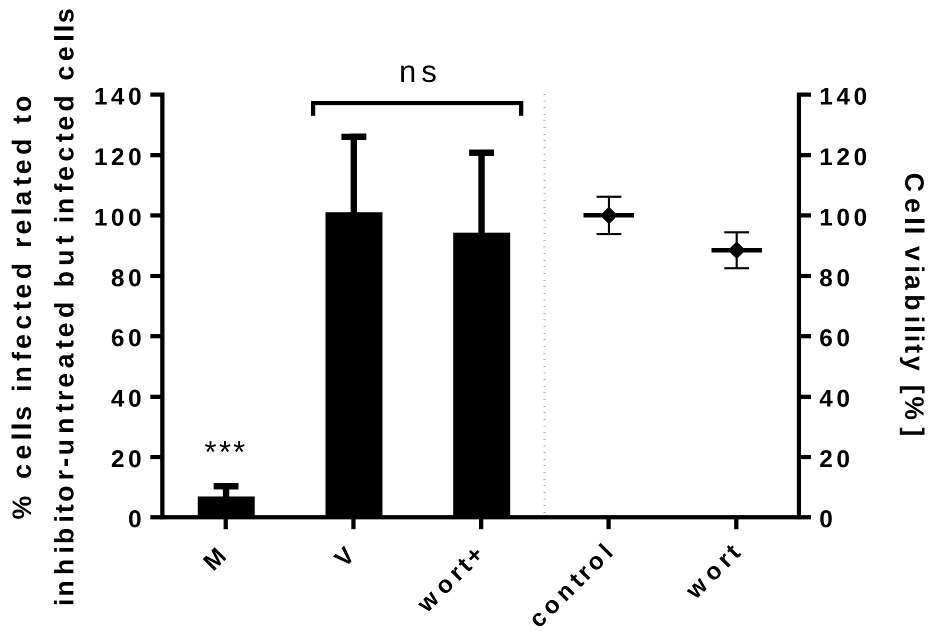

Supplementary Figure 3. HCoV-OC43 replication in HCT-8 cells in the presence of micropinocytosis inhibitors. HCT-8 cells were pre-treated with wortmannin, infected with HCoV-OC43 and analysed by flow cytometry 3 days p.i. The infection efficiency determined with flow cytometry is expressed as the percentage of HCoV-OC43 infected cells, compared to the untreated control, and is presented on the left side of the graph. Right part of the graph shows the cell viability, as determined with an XTT assay. wort – 5 μM wortmannin; control – PBS treated cells; M – mock infected cells; V or + – HCoV OC43 infected cells. The data is presented as the mean of a triplicate for each sample ± SD. To determine the significance of differences between compared groups, Single-Factor Analysis of Variance (ANOVA) was applied. *** P values <0.05 were considered significant.

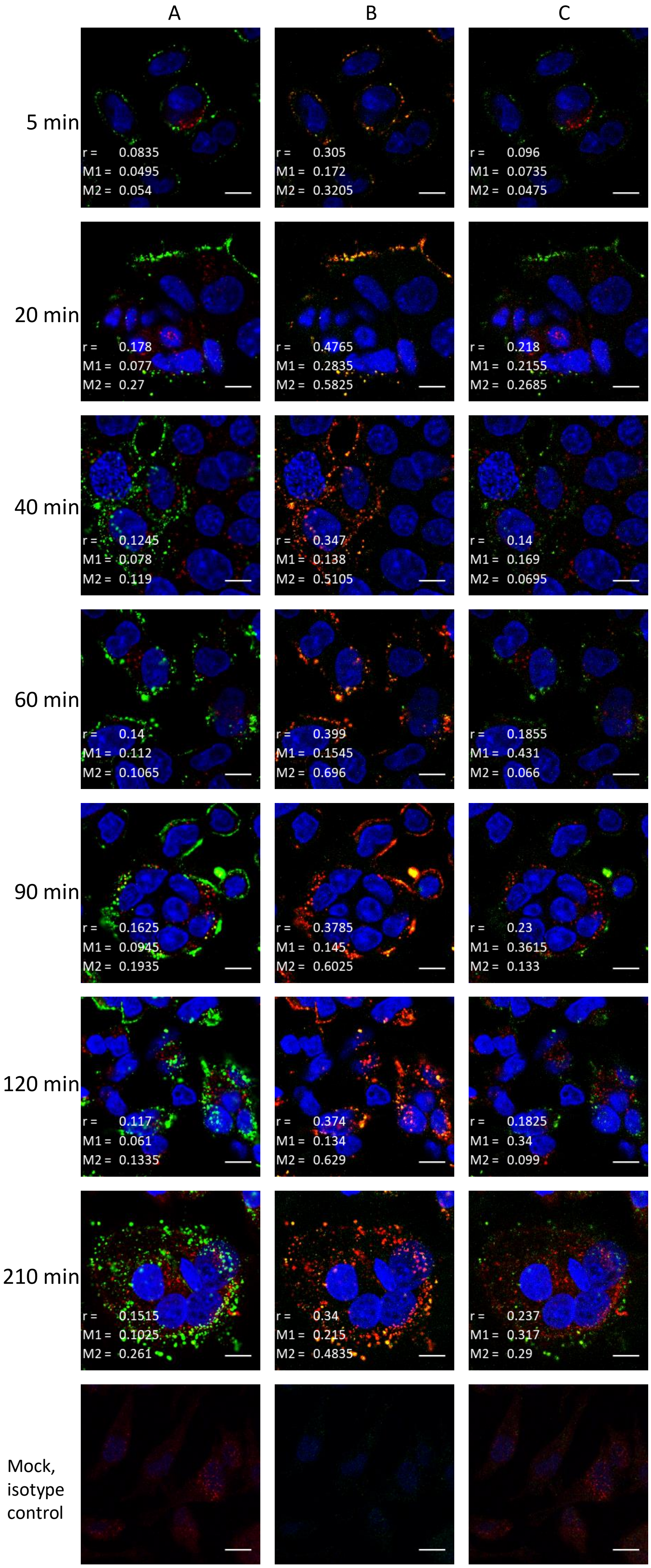
A
B
C
5 min
r = 0.0835
M1 = 0.0495
M2 = 0.054
r = 0.305
M1 = 0.172
M2 = 0.3205
r = 0.096
M1 = 0.0735
M2 = 0.0475
20 min
r = 0.178
M1 = 0.077
M2 = 0.27
r = 0.4765
M1 = 0.2835
M2 = 0.5825
r = 0.218
M1 = 0.2155
M2 = 0.2685
40 min
r = 0.1245
M1 = 0.078
M2 = 0.119
r = 0.347
M1 = 0.138
M2 = 0.5105
r = 0.14
M1 = 0.169
M2 = 0.0695
60 min
r = 0.14
M1 = 0.112
M2 = 0.1065
r = 0.399
M1 = 0.1545
M2 = 0.696
r = 0.1855
M1 = 0.431
M2 = 0.066
90 min
r = 0.1625
M1 = 0.0945
M2 = 0.1935
r = 0.3785
M1 = 0.145
M2 = 0.6025
r = 0.23
M1 = 0.3615
M2 = 0.133
120 min
r = 0.117
M1 = 0.061
M2 = 0.1335
r = 0.374
M1 = 0.134
M2 = 0.629
r = 0.1825
M1 = 0.34
M2 = 0.099
210 min
r = 0.1515
M1 = 0.1025
M2 = 0.261
r = 0.34
M1 = 0.215
M2 = 0.4835
r = 0.237
M1 = 0.317
M2 = 0.29
Mock, isotype control

Supplementary Figure 4. Co-localization of HCoV-OC43, early endosomes' marker EEA1 and dextran. Co localization of HCoV-OC43, EEA1 and dextran in HCT-8 cells at different time points post-infection was studied with confocal microscopy. Respective time points are indicated on the left side of the figure. Co-localization of HCoV-OC43 (green) with EEA1 (red) is shown in column A. Column B presents co-localization of HCoV-OC43 (green) with dextran conjugated to tetramethylrodamine (dextran-TMR; red). In column C co-localization of dextran-TMR (red) with EEA1 (green) is shown. Nuclei are presented in blue. Scale bar = 10 μm. Co-localization parameters: r – Pearson's coefficient; M1 - Manders' coefficient M1 (red overlapping green); M2 - Manders' coefficient M2 (green overlapping red). The experiment was conducted at least thrice, and representative images are presented.
